# Degradation of Fatty Acid Phase-Change Materials (PCM): New Approach for Its Characterization

**DOI:** 10.3390/molecules26040982

**Published:** 2021-02-12

**Authors:** Marc Majó, Ricard Sánchez, Pol Barcelona, Jordi García, Ana Inés Fernández, Camila Barreneche

**Affiliations:** 1 Department of Materials Science and Physical Chemistry, Universitat de Barcelona, Martí i Franqués 1, 08028 Barcelona, Spain; marc.majo@ub.edu (M.M.); ricardsanchezvalls@gmail.com (R.S.); pbarcelona94@ub.edu (P.B.); ana_inesfernandez@ub.edu (A.I.F.); 2 Departament de Química Inorgànica i Orgànica, Secció de Química Orgànica, Universitat de Barcelona, Martí i Franqués 1, 08028 Barcelona, Spain; jordigarciagomez@ub.edu

**Keywords:** thermal energy storage, phase change materials, fatty acids, degradation, DSC, TGA, GC, NMR, rheology, materials characterization

## Abstract

The use of adequate thermal energy storage (TES) systems is an efficient way to achieve thermal comfort in buildings reducing the cooling and heating demand. Besides, deploy phase change materials (PCM) to meet and enhance the TES needs is highly effective and widely studied. In this paper, a study of the degradation of two fatty acids is presented, capric and myristic acids, in order to evaluate whether their thermo-physical properties are affected throughout time during service. This was carried out by means of two different types of thermal treatments: degradation at constant temperature (thermal stability test), 60 °C during 100 h and 500 h, and degradation with heating and cooling cycling (thermal cycling stability), between a temperature range from 15 °C to 70 °C with 0.5 °C/min ramp during 500 and 1000 cycles. Despite no significant changes were measured for myristic acid, experimental results revealed a decrease of melting enthalpy of 6.6% in capric acid thermally treated for 500 h. Evidences of chemical degradation were found that might explain the decrease in thermophysical properties during use.

## 1. Introduction

High fossil fuel dependence as a main energy resource has created an energetic-global problem since the economic, industrial and demographic developments has been based on this resource. Within the energy sector, CO_2_ resulting from the combustion of carbon fuels dominates the emission of greenhouse gasses. It accounts for the largest share of global anthropogenic greenhouse emissions, representing about 58% of global emissions [1]. 

In the building sector, heating, ventilation and air conditioning systems (HVAC) represents an important part of the global energy demand, reaching 36% of energy consumption worldwide [2,3]. In order to address this main problem, the use and development of renewable energy sources is required as well as the improvement of the energy efficiency systems in buildings. One of the most promising technologies in order to enhance the energy efficiency of this sector are thermal energy storage (TES) systems. The use of TES system can contribute with an increasing of the overall efficiency and reliability as well as better economic feasibility, less pollution and low CO_2_ emissions [4]. 

Thermal energy storage in buildings can be implemented by sensible heat, raising the temperature of a solid or liquid, or by latent heat, absorbing or releasing heat as it undergoes a phase change (solid to solid or solid to liquid). The main advantage of latent heat storage (LHS) is the high storage density in small temperature range [5]. Moreover, different technologies can be implemented: On one side, passive TES technology, which has the objective to provide thermal comfort with the minimum use of HVAC energy, storing high quantity of energy giving thermal stability inside the building. On the other hand, active TES technology, which is an excellent complement with HVAC systems or renewable energy sources (water heating, ventilation systems, passive solar architecture or PV panels) to increase the efficiency of the existing HVAC methods [6]. 

The use of Phase Change Materials (PCM) in order to fulfil and enhance the TES systems is widely known. Today, large number of PCMs can be implemented as TES such as inorganic, organic or mixtures. Among all investigated PCMs, fatty acids possess many superior properties: wide range of melting temperature suitable for different applications, high heat capacity, low vapor pressure, non-toxic and non-corrosive behaviour, notable chemical and thermal stability, small volume change during the phase transition, non-flammability and specially, low cost [7]. Fatty acids also show reproducible melting and freezing behaviour with no supercooling [8]. However, one of the main limitations in order to apply fatty acids PCM in thermal energy storage lies in their low thermal conductivity. Therefore, several heat transfer enhancement methods, such as innovative fin configurations [9,10], metal foams [11] and high-heat-conductivity nanoparticles [12], have been studied towards an improvement on thermal performance of LHS. 

Besides that, the degradation of the compound after a large number of melting and solidification cycles, is an important fact to take into account. Hence, this paper is focused in the study of degradation of two fatty acids used as PCM, capric acid (C_10_H_20_O_2_) and myristic acid (C_14_H_28_O_2_), in order to evaluate whether their composition and thermo-physical properties are affected with temperature fluctuations during operational time. This will be performed with two different types of thermal treatments.

The degradation of the PCM is related to a significant change on the structure and properties of the molecule due to large number of thermal cycles or phase transitions. This could lead to a chain scission, ramification or oxidation of the material that would change its thermal advantages. Previous studies have dealt with this problem through infrared spectroscopy (IR) analysis. Nevertheless, due to thermo-oxidative degradation there is no significant difference in the functional groups identified after the degradation of the acid and, consequently, the evaluation of this degradation by means of FT-IR does not provide relevant information. To handle this issue, it is proposed to evaluate the viscosity of the PCMs with a different time of thermal exposure, and to use chemical analysis methods such as gas chromatography (CG) and nuclear magnetic resonance (MNR) to assess the formation of new compounds due to degradation. The impact of the chemical degradation on the thermophysical properties is assessed with thermogravimetric analysis (TGA) and the measurement of the thermal properties through Differential Scanning Calorimetry (DSC). 

## 2. Results

### 2.1. Changes in Chemical Composition or Chemical Structure

**Figure 1:**
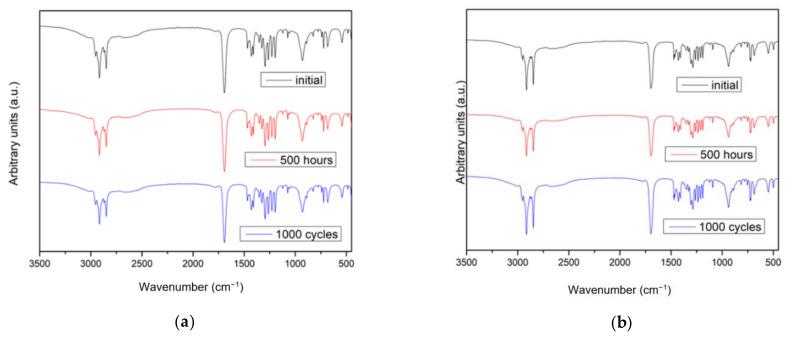
FT-IR results: (**a**) capric acid samples: initial, thermal degradation 500 h, and thermal cycling 1000 cycles (**b**) myristic acid: initial, thermal degradation 500 h, and thermal cycling 1000 cycles.

Based on the FT-IR spectra obtained from the samples with the two methods of degradation, a comparison of the results is not feasible to verify if a degradation process has occurred in the fatty acid samples. Although a similar result is attributed to the absence of changes in the chemical structure of studied fatty acids, and thus the fatty acids do not degrade during thermal cycles, we disagree with this statement. During the degradation some reactions such as ramification or polymerisation can take place, and these changes will not be reflected with FT-IR, as it does not reflect the presence of degradation products with new peaks, since no new functional groups are detected. The FT-IR spectra of both fatty acids after the worse conditions of thermal treatments (500 h at 60 °C and 1000 cycles) are shown in Figure 1. It is possible to see around 3000 cm^−1^ the peaks corresponding to C-H stretching and the peak recorded at about 1715 cm^−1^ the one related to C=O stretching signal [13]. Capric acid and myristic acid only differ in the number of carbons their molecular chain has; therefore, the FT-IR spectra are very similar. 

**Figure 2:**
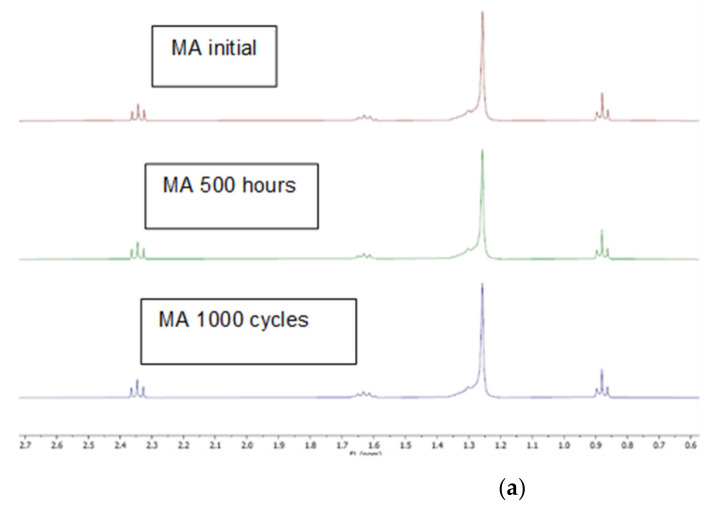

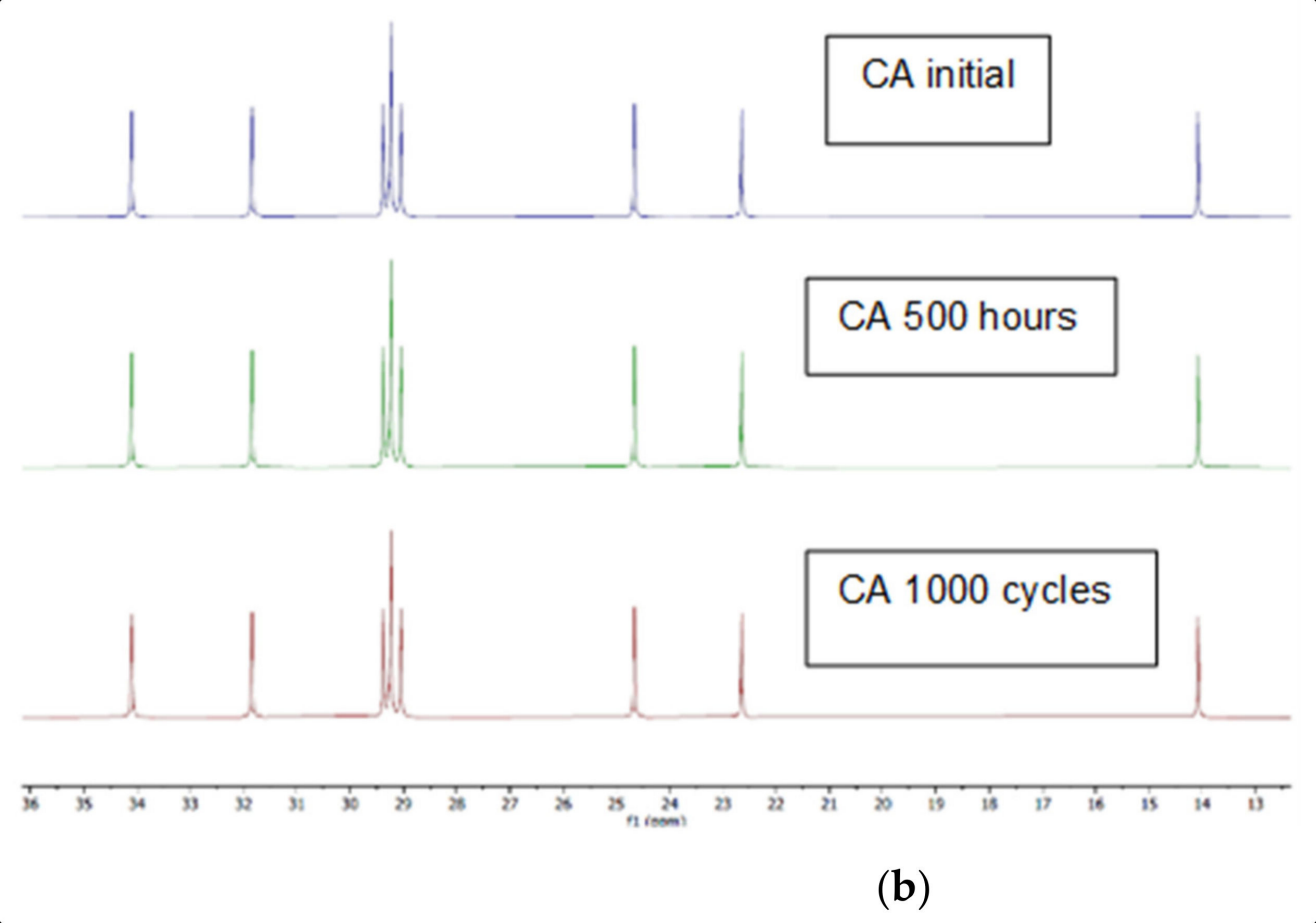
NMR results: (**a**) ^1^H spectra of myristic acid samples (**b**) ^13^C spectra of capric acid samples.

The analysis of the ^13^C and ^1^H spectra of initial samples, samples treated during 500 h at 60 °C and samples with 1000 thermal cycles for capric acid and myristic acids does not reveal significant differences between them. As can be seen in Figure 2, the ^1^H spectra for the myristic acid samples analysed have the same profile, and a similar result was obtained for capric acid samples; meaning that samples have the same composition within the detection limits of this technique. The thermo-oxidative processes involve radical reactions that may lead to chain scission and further ramification of the chains. Depending on the amount of ramifications produced it is expected a differentiate signal for the tertiary carbons in a ^13^C spectrum. However, the analysis of initial and treated samples does not show significant differences, as can be seen in Figure 2 in the example for capric acid samples. Similar results were obtained for myristic acid samples, revealing that if ramification took place, its extension is below the detection of limit of NMR ^13^C, estimated around 5%. 

**Figure 3:**
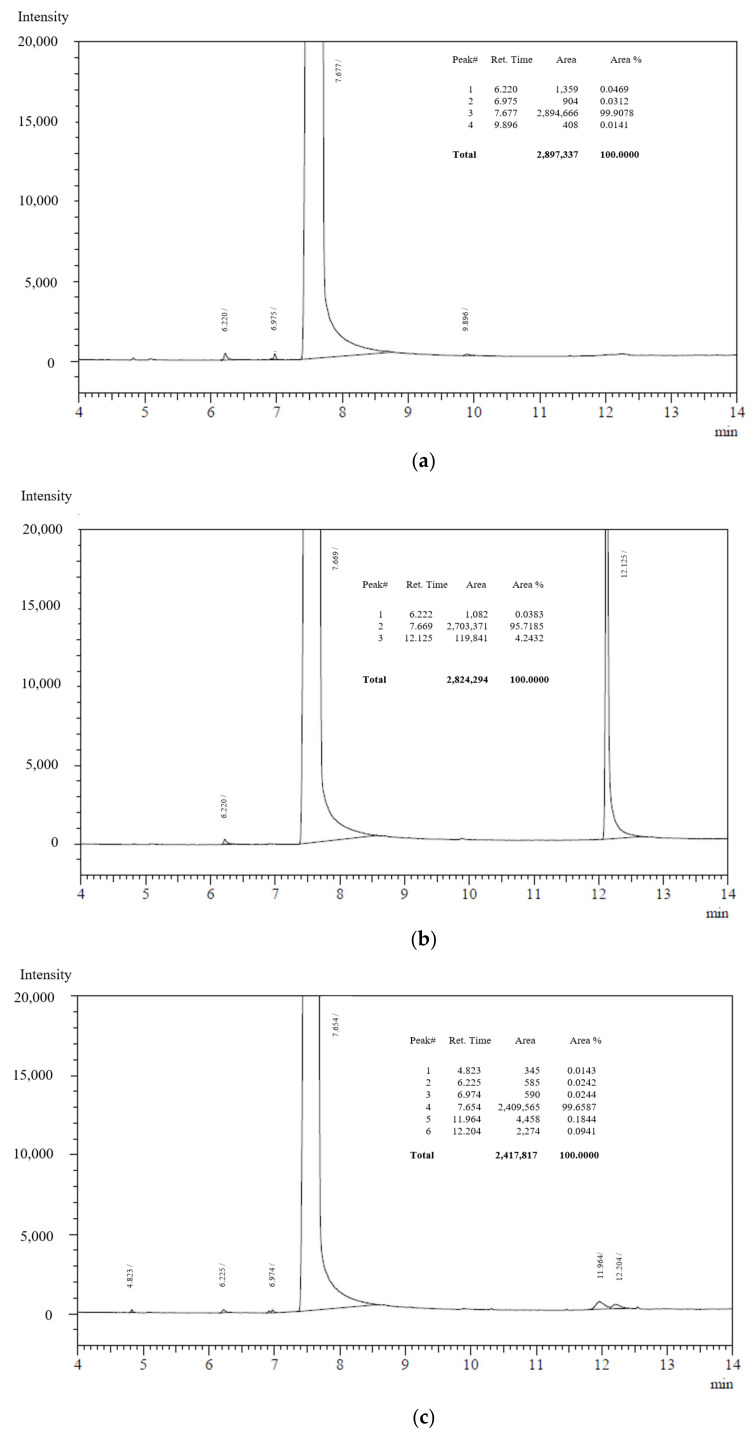
CG results: (**a**) capric acid, initial (**b**) capric acid thermal degradation, 500 h (**c**) capric acid thermal cycling degradation, 1000 cycles.

The evidence of the existence of degradation products after the thermal treatments was found using a separation strategy with Gas Chromatography (GC). Results between capric acid samples and myristic acid samples showed differences; despite no evidence of degradation products was observed in the chromatograms of myristic acid and both thermal treated (500 h) and thermal cycled samples, different results were obtained for capric acid samples. As can be seen in Figure 3, the initial capric acid sample is a quite pure compound (integrated area 99.9%), with a low concentration of impurities of smaller molecular weight. These impurities are seen as the small peaks appearing around 6 min retention time. 

The thermal treatment at 60 °C for 500 h had a significant effect on the acid capric sample and GC shows a degradation product at 12.125 min retention time. In addition, while the peak at 6220 min attributed to an impurity in the initial sample also appeared in this treated sample, it does not happen with the small peak seen at 6975 min. Peaks at higher retention times in GC are associated at more polar and/or higher mass products. Indeed, these results suggest that chain scission and reaction between formed radicals took place in some extension, producing longer chains and thus bigger carboxylic acid molecules. The integrated peak area for this compound is 4.2%, explaining why it is not possible to identify this degradation product with NMR because of its resolution. 

Result for the thermal cycled sample (1000 cycles) is better in terms of degradation products formation. This thermal treatment led to the formation of different degradation products, as shown by the small peaks in the chromatogram at 11.962 min and 12.204 min retention time with a very small concentration. An additional small peak at 4823 min is also identified, that was not in the initial sample. 

**Figure 4:**
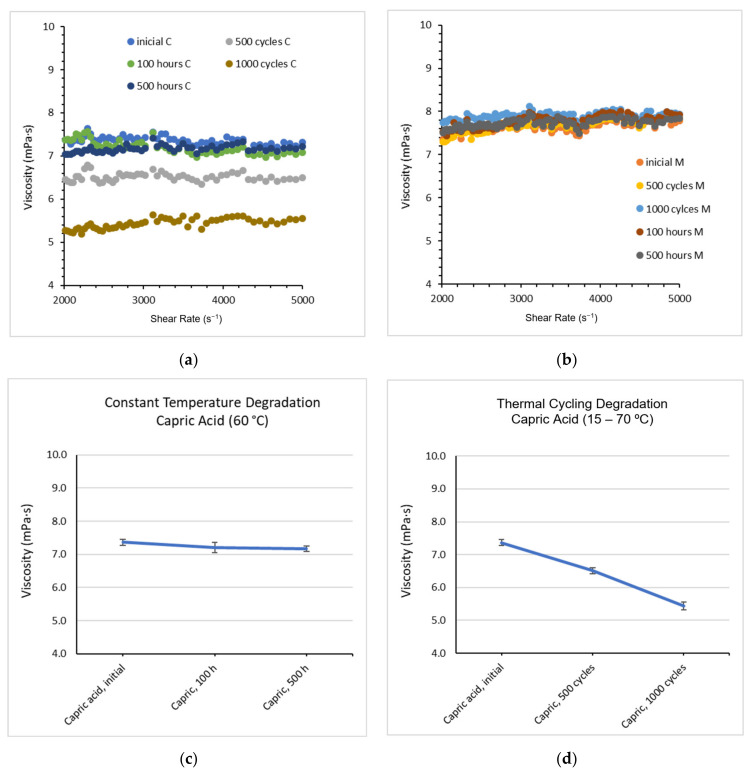

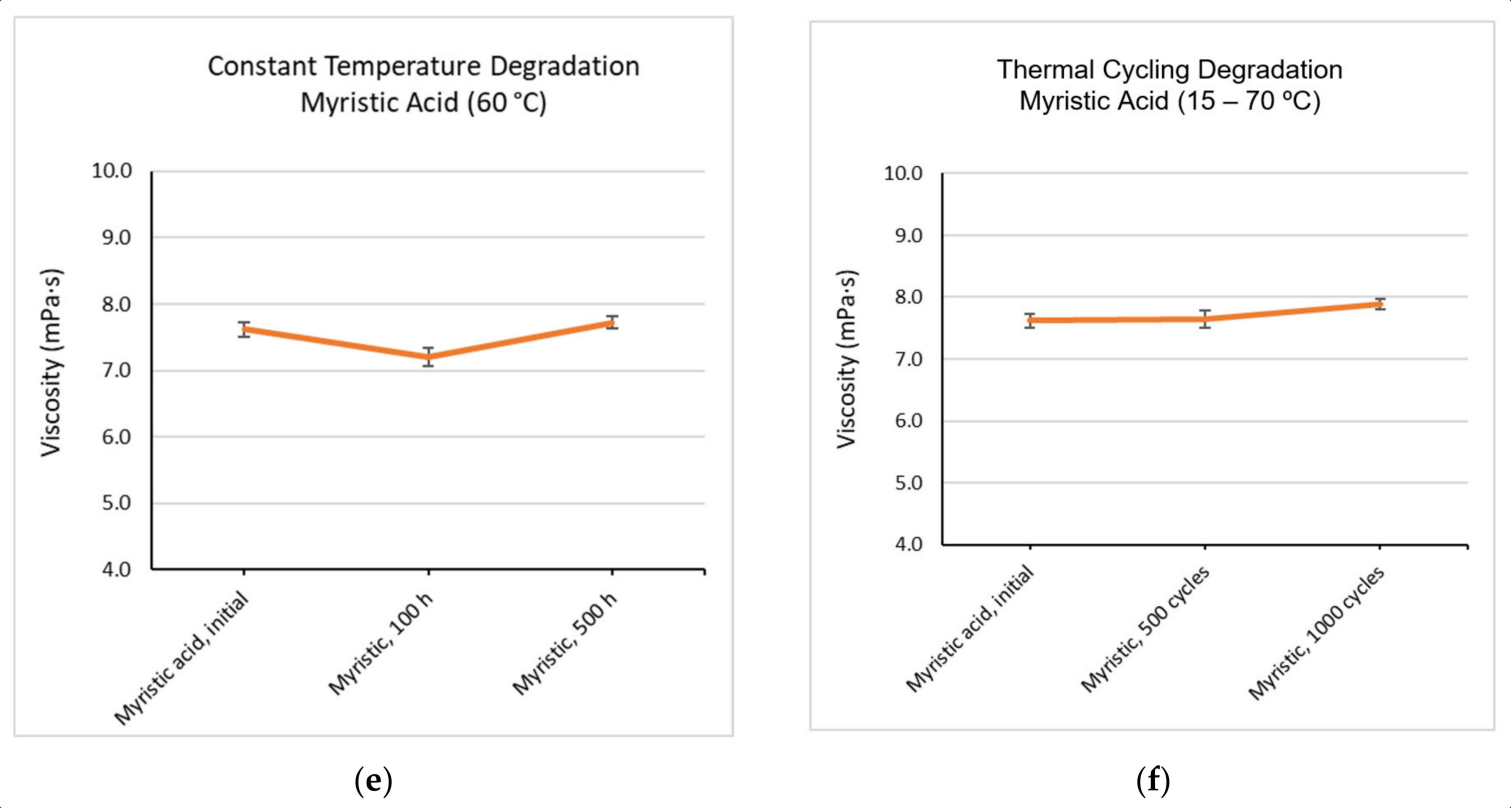
Viscosity curves of (**a**) capric acid samples at 35 °C, (**b**) myristic acid samples at 60 °C. Variation of viscosity for (**c**) capric acid thermal degradation, (**d**) capric acid thermal cycling degradation, (**e**) myristic acid thermal degradation, (**f**) myristic acid thermal cycling degradation.

Figure 4a,b shows the viscosity of the capric and myristic acids under study respectively. Given the low viscosity of the samples, only the segment between 2000 and 5000 s^−1^ is relevant since the lowest strain rates are outside the measuring range of the used cone. All samples show a prevailing Newtonian behaviour. 

Capric acid presents an initial viscosity of 7.36 mPa·s (35 °C). Figure 4c shows that despite the presence of a degradation product of higher molecular weight, the thermal degradation at constant temperature does not noticeably change the capric acid viscosity, with minimal variation thought test time. However, the thermal cycled samples showed a significant change in the final viscosity of a 35% at 1000 cycles, as can see in Figure 4d. This change can be directly associated with the degradation of the material, forming other components of shorter structure that facilitate the fluidity of the material.

Myristic acid show a viscosity of 7.62 mPa·s (60 °C). There are not significant changes in the viscosity of the myristic acid with different thermal treatments, which is in accordance with the results of chemical characterization. 

**Figure 5:**
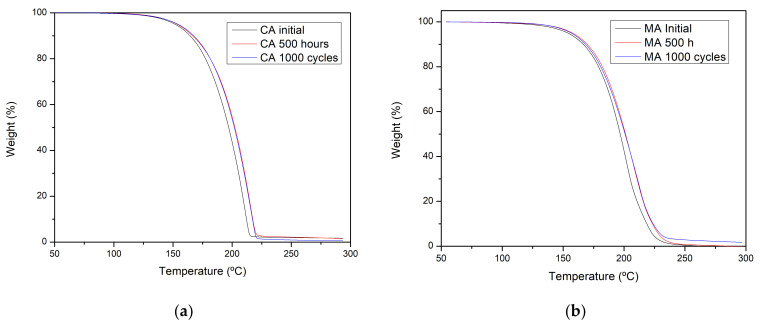
TGA results: (**a**) Capric acid thermal and cycling stability, (**b**) Myristic acid thermal and cycling stability.

The effect of thermal treatments on the thermal stability was evaluated with TGA, which gives information related to the maximum working and degradation temperature of the samples under study. As can be seen in Figure 5, there are not significant differences in the thermal decomposition of the samples despite the presence of small amount of impurities, and the degradation product observed for the thermal treated capric acid sample does not affect the thermal degradation. 

### 2.2. Latent Heat Stored

**Figure 6:**
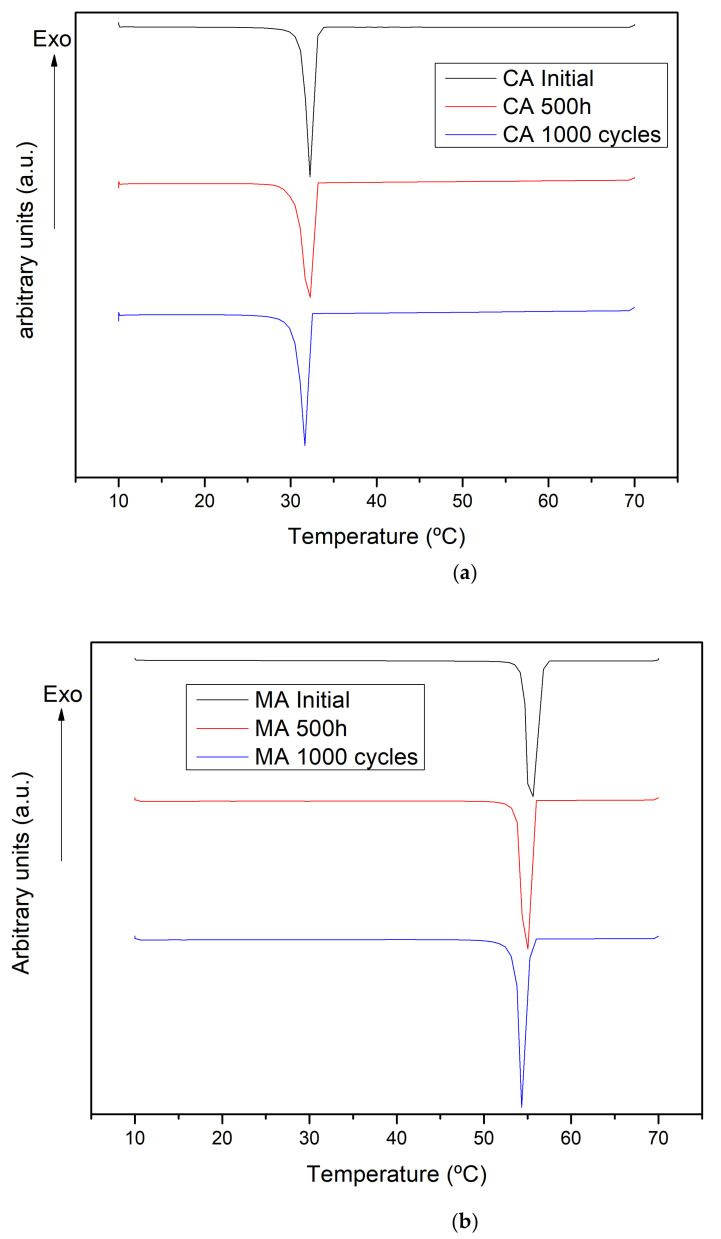
DSC results: (**a**) Capric acid: initial, thermal stability 500 h, cycling stability 1000 cycles (**b**) Myristic acid initial, thermal stability 500 h, cycling stability 1000 cycles.

Figure 6 shown the results obtained by DSC and Table 1 summarizes values of melting enthalpy and temperature. These results compare the initial samples with the thermal treated samples under extreme conditions, which are 500 h at 60 °C and after 1000 thermal cycles. The results here presented are the mean obtained by 3 repetitions. There is a general trend for both fatty acids; the melting point is slightly reduced after the thermal treatments, less than 2% for all the samples tested. As a change of few degrees regarding the phase change temperature could be crucial when selecting a PCM, it is important to know how this property changes after PCM degradation. 

The melting enthalpy follows the same trend decreasing for all the samples thermal treated, with greater reduction, 6.6%, measured for the capric acid after 500 h at 60 °C, the most degraded sample according with the chemical characterization. This fact suggests a cryoscopic effect due to the presence of impurities. Therefore, the thermal storage capacity is slightly affected by degradation process when these PCM are thermal treated. 

As Sari et al. mention in their publication [14], it is recommended that before using fatty acids as PCM, they must be tested under accelerated thermal cycle test in order to understand how the material will thermally behave.

In addition, the differences with the melting enthalpy values reported by NIST Standard References Database [15] and the values obtained in this study are due to the differences in the conditions to measure the melting enthalpy. Lazaro et al. [16] described that the melting enthalpy measured by DSC highly depends on the method applied, the DSC equipment used and the amount of sample used to perform the analyses. Barreneche et al. described that the enthalpy results obtained for materials to be used as phase change materials will depend on the method programed in the DSC [17].

## 3. Materials and Methods

### 3.1. Materials and Thermal Treatments

The PCM used are the fatty acids capric acid (C_10_H_20_O_2_) and myristic acid (C_14_H_28_O_2_) from Merck Life Science, and their purity was ≥98% (food grade). Mostly impurities in fatty acids typically are moisture or insoluble matter, however, some fatty acids with similar length, could be spotted in the product. 

Two different methods of degradation were studied: degradation at constant temperature throughout time (thermal stability test), using glass beakers as containers, and degradation with heating and cooling cycling (thermal cycling stability), using polyethylene Eppendorf like tubes as containers. The first method used a thermal bath at 60 °C during 100 h and 500 h for each acid. In addition, the thermal cycling test was performed in a range of temperature from 15 °C to 70 °C with 0.5 °C/min ramp and checking the composition and thermophysical properties after 500 and 1000 cycles for each type of acid. 

### 3.2. Fatty Acid Characterization

The chemical groups characterization of the fatty acids was performed by Fourier transformed infrared (FT-IR) spectroscopy in order to analyse the chemical bond vibration providing several characteristic peaks of each molecular bond. Attenuated total reflectance (ATR) FT-IR instrument Spectrum Two ^TM^ from Perkin Elmer (Waltham, MA, USA) was used to perform the analysis. 

Nuclear magnetic resonance NMR of ^1^H and ^13^C was performed on some of the samples dissolved in CDCl_3_, searching evidences of chemical changes such as chain scissions, ramification and oxidation. The measurements were carried out with a Bruker BioSpin GmbH B400 (Billerica, MA, USA). 

Gas Chromatography GC Shimadzu QP2010 (Kyoto, Japan) was used to identify de presence of degradation products. Thus, a column HP-5 (5%PH ME Polysiloxane), with He as carrier gas was used. The initial temperature was 100 °C (hold time 1 min), and the final temperature 240 °C (hold time 3 min) with a heating rate of 10 °C/min and the injection of 2 microliters of samples of ~2 mg/mL in MeOH. 

The viscosity of the fatty acids was measured by a rheometer Brookfield RST-CPS (Middleborough, MA, USA) with a Peltier control temperature device. The measurement was carried out at 35 °C and 60 °C for capric and myristic acid samples respectively. A lineal ascendant ramp of shear rate from 1000 to 5000 s^−1^ in control by shear rate mode (CSR) has been carried out using a cone plate geometry of 50 mm of diameter, with a cone angle of 1° and a truncation of 50 µm.

The thermal stability of the materials under study were analysed by thermogravimetric analysis (TGA). This technique is used to determinate the degradation of the materials under study by the measurement of the weight loss of a sample under a temperature increment in a controlled atmosphere. This analysis is performed in order to determinate the upper service temperature of both fatty acids. The instrument used is a TGA/DSC DSTQ600 from TA Instruments (New Castle, DE, USA). The analysis was performed in a range of temperature from 50 °C to 300 °C with 10 °C/min heating rate under a constant 50 mL/min synthetic air. 

The heat storage capacity of the PCM here studied were analysed by differential scanning calorimetry (DSC). DSC is a powerful technique to measure thermophysical properties of a substance such as latent heat or specific heat capacity as well as phase change temperature. Dynamic mode was used as execution mode. It is based on a 0.5 °C/min heating constant rate with the same procedure in cooling constant rate [13]. The equipment used was a DSC 822e Star3+ from Mettler Toledo (Ciudad de México, México). The experiments were performed in a temperature range from 10 °C to 70 °C with 0.5 °C/min heating-cooling rate and 50 mL/min N_2_ flow. The amount of sample characterized was around 15 mg, and the samples were placed inside 40 μL aluminium crucibles. The calibration of DSC used in this study is performed by applying an algorithm correction when 2 different patterns are measured under the standard conditions defined by the supplier (Zn and Ga). The external calibration of the DSC shows that the error for the melting temperature is 0.1 °C and the error for the enthalpy is 3 J/g. This values are common in DSC equipment.

## 4. Conclusions

Capric and myristic acid thermal treatments were produced through two different procedures, in a thermal bath at 60 °C and by thermal cycling the samples between 15 °C and 70 °C. Chemical characterization of degradation products were studied with FT-IR, NMR and GC the last being the only useful technique to detect degradation products, and only in the capric acid samples, but not in the myristic acid ones. Moreover, a degradation product with higher molecular weight than capric acid, with a concentration around 4% was detected for the sample thermal treated for 500 h. This treatment also produced the highest decrease of melting enthalpy, 6.6%, while slight changes were also measured for all the treated samples, as well as slight decreases in melting enthalpy. Moreover, the study of thermal stability with TGA does not reveal significant changes due to degradation. Evidences of degradation of capric acid samples were also found with the rheological characterization that showed significant changes in thermal cycled capric acid sample that may be produced by the presence of degradation compound of lower molecular weight. 

Therefore, these results confirm that degradation in fatty acids takes place in smaller molecules in higher extension. Even though small changes in melting enthalpy and melting temperature can be measured, the detection and identification of degradation products is a difficult task with chemical characterization methods such as FT-IR or NMR because of the detection limits. 

## Figures and Tables

**Table 1:** DSC results: melting temperature and melting thermal and cycling stability results. ΔH_m_[%] are melting enthalpy variation expressed in % regarding the initial value and ∆T_m_ [°C] are temperature increment in the different treatments.

Sample	N° Hours/Cycles	ΔH_m_ [J·g^−1^]	ΔH_m_ [%]	T_m_ [°C]	ΔT_m_ [°C]
Capric acid	Initial	159 ± 1		32.3 ± 0.0	
	500 h	149 ± 1	−7	32.1 ± 0.0	−0.19
	1000 cycles	153 ± 1	−4	31.8 ± 0.0	−0.46
Myristic acid	Initial	190 ± 1		55.3 ± 0.0	
	500 h	182 ± 1	−4	54.6 ± 0.0	−0.66
	1000 cycles	184 ± 1	−3	54.6 ± 0.0	−0.74

## Data Availability

The data is available by demand.

## Sample Availability

Samples of the compounds presented in this work are available from the authors and will be kept in our labs during 12 months after the publication.

## References

[1] International Energy Agency (IEA) CO_2_ emissions from fuel combustion. https://webstore.iea.org/download/tableofcontents/5.

[2] International Energy Agency (IEA) Total Energy Consumption 2020. https://yearbook.enerdata.net/total-energy/world-consumption-statistics.html.

[3] Diarce G., Campos-Celador Á., Martin K., Urresti A., García-Romero A., Sala J. (2014). A comparative study of the CFD modeling of a ventilated active façade including phase change materials. Appl. Energy.

[4] Navarro L., De Gracia A., Colclough S., Browne M., McCormack S.J., Griffiths P., Cabeza L.F. (2016). Thermal energy storage in building integrated thermal systems: A review. Part 1. active storage systems. Renew. Energy.

[5] Cabeza L.F., Castell A., Barrenechea C., de Gracia A., Fernández A.I. (2011). Materials used as PCM in thermal energy storage in buildings: A review. Renew. Sustain. Energy Rev..

[6] De Gracia A., Cabeza L.F. (2015). Phase change materials and thermal energy storage for buildings. Energy Build..

[7] Yuan Y., Zhang N., Tao W., Cao X., He Y. (2014). Fatty acids as phase change materials: A review. Renew. Sustain. Energy Rev..

[8] Sharma A., Tyagi V.V., Chen C.R., Buddhi D. (2009). Review on thermal energy storage with phase change materials and applications. Renew. Sustain. Energy Rev..

[9] Zhang C., Li J., Chen Y. (2020). Improving the energy discharging performance of a latent heat storage (LHS) unit using fractal-tree-shaped fins. Appl. Energy.

[10] Sheikholeslami M., Lohrasbi S., Ganji D.D. (2016). Numerical analysis of discharging process acceleration in LHTESS by immersing innovative fin configuration using finite element method. Appl. Therm. Eng..

[11] Deng Z., Chen Y., Cheng-Bin Z., Huang Y., Chen Y.-P. (2017). Melting behaviors of PCM in porous metal foam characterized by fractal geometry. Int. J. Heat Mass Transf..

[12] Barreneche C., Martín M., La Rosa J.C.-D., Majó M., Fernandez A. (2019). Own-Synthetize Nanoparticles to Develop Nano-Enhanced Phase Change Materials (NEPCM) to Improve the Energy Efficiency in Buildings. Molecules.

[13] Karaipekli A., San A. (2009). Capric–myristic acid/vermiculite composite as form-stable phase change material for thermal energy storage. Sol. Energy.

[14] Sari A., San H., Onal A., Sari A. (2004). Thermal properties and thermal reliability of eutectic mixtures of some fatty acids as latent heat storage materials. Energy Convers. Manag..

[15] Acree W., Chickos J.S. (2010). Phase Transition Enthalpy Measurements of Organic and Organometallic Compounds. Sublimation, Vaporization and Fusion Enthalpies From 1880 to 2010. J. Phys. Chem. Ref. Data.

[16] Lazaro A., Peñalosa C., Solé A., Serras P., Haussmann T., Fois M., Zalba B., Gschwander S., Cabeza L.F. (2013). Intercomparative tests on phase change materials characterisation with differential scanning calorimeter. Appl. Energy.

[17] Barreneche C., Solé A., Miró L., Martorell I., Fernandez A., Cabeza L.F. (2013). Study on differential scanning calorimetry analysis with two operation modes and organic and inorganic phase change material (PCM). Thermochim. Acta.

